# Preoperative Patient Preparation Audit: Completed Loop Cycle

**DOI:** 10.7759/cureus.4155

**Published:** 2019-02-28

**Authors:** MN Baig, Conor Keady, Sandra O'Malley, C M Hurley, Evelyn Murphy

**Affiliations:** 1 Orthopaedics, University Hospital Galway, Galway, IRL; 2 Plastic Surgery, University Hospital Galway, Galway, IRL; 3 Orthopedics, Cappagh National Orthopaedic Hospital, Dublin, IRL

**Keywords:** checklist, pre-operative

## Abstract

Introduction

Nurses usually check patients scheduled for surgery while the patients are still in the ward. A lack of complete preoperative patient preparation can cause delayed care and disastrous outcomes. The objective of this study was to assess the number of patients en route to surgery who had been fully preoperatively prepared and evaluate any change in that number once a proforma was introduced as part of the preparation protocol.

Methods

We conducted a two-part audit of preoperative preparedness to assess factors such as up-to-date blood work, group and save, cross-match, and surgical site marking, among others. We then devised a proforma to be signed and checked by the ward doctor (e.g., intern or senior house officer). We compared the number of patients marked completely ready for surgery in the six weeks prior to use of the proforma with the number of patients marked completely ready for surgery for six weeks after implementation of the proforma.

Results

The study included the preoperative audit of 35 patients prior to the use of the proforma and 30 patients after the implementation of the proforma. Use of the proforma improved preoperative patient preparation by 50% compared to the level of preparedness when no proforma was used.

Conclusion

Health care facilities may benefit from a similar proforma for supplementing standardized, widely accepted preoperative protocols as an additional safety measure.

## Introduction

Audits are an important tool in improving clinical practice [1]. Time in the operating theatre is a costly limited resource with little margin for error. Therefore, efficiency is crucial for good outcomes. Most surgical facilities in Western medicine follow the World Health Organization (WHO) checklist for preparing patients for surgery [2-3]. The WHO checklist covers the confirmation of the patient’s identity, surgical site, patient allergies, the surgical procedure, theater preparation, and anesthetic preparation. The checklist is a tool to help health care professionals anticipate potential outcomes and encourage the timely and efficient use of surgical resources.

Despite the safeguards represented by the WHO checklist, some patients proceed to surgery without fully and appropriate preoperative preparation. Without proper preoperative preparation, patients and health care facilities are placed at risk for a myriad of poor outcomes, medically and legally. We conducted this study to assess the number of patients en route to surgery who had been fully preoperatively prepared and evaluate any change in that number once a proforma was introduced in addition to WHO surgical theatre checklist [4].

## Materials and methods

**Figure 1:**
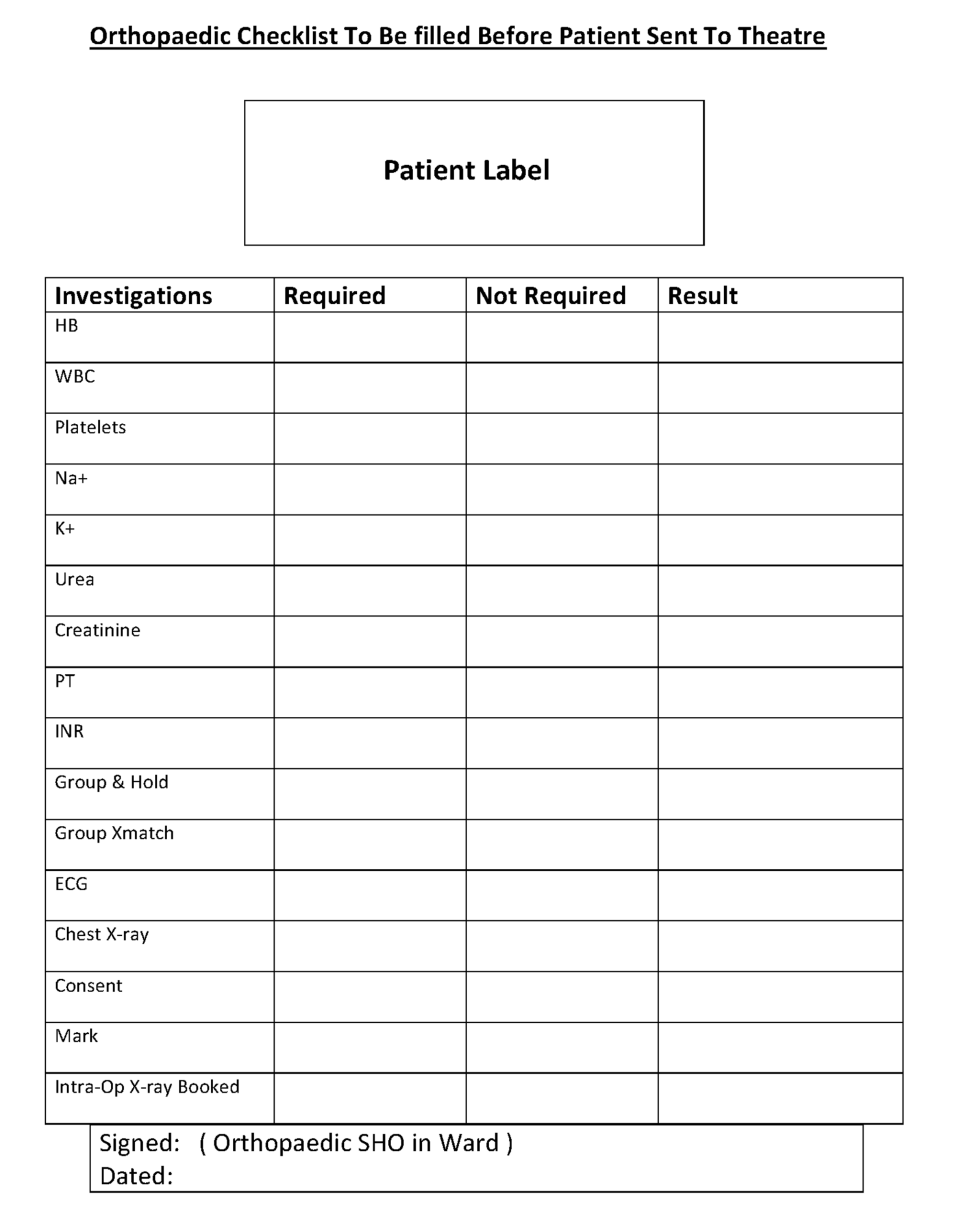
Proforma developed for the second audit This form was made to be completed by the ward SHO before the patient was transferred to the theatre. HB: Haemoglobin; WBC: White blood cell; NA+: Sodium; K+: Potassium; PT: Prothrombin time; INR: International normalised ratio; ECG: Electrocardiogram; SHO: Senior house officer.

The first part of our two-part audit was conducted over six weeks in July and August 2018 to assess the preparedness of 35 patients prior to surgery using our standard facility protocols. The second part of the audit was conducted over six weeks after implementing a proforma to be completed and signed by the orthopaedic ward senior house officer (SHO) before patients were sent to the operating theatre (Figure 1). The proforma’s development was informed by the shortcomings discovered during the first part of the audit.

## Results

The results of the first six-week audit are presented in Table 1. Eleven of the 35 patients experienced preparation errors resulting in delayed surgical operations. Table 2 presents the results of the second six-week audit conducted after implementing the proforma to be signed by the SHO before sending patients to the operating theater. Of 30 patients prepared for a surgical operation, five patients experienced an error in preparation that caused delay. Table 3 shows the improvement in patient preparation from the first audit to the second audit.

**Table 1:** Audit of preoperative errors before use of the proforma 35 patients included over six weeks.

Error Type	n=35
Missing up-to-date blood work	2
Missing group and hold/cross-matching	1
Surgical site not marked	2
Consent form not signed	1
Theatre x-ray scheduling conflict	5

**Table 2:** Audit of preoperative errors after the use of the proforma 30 patients over six weeks.

Error type	n= 30
Missing up-to-date blood work	1
Missing group and hold/cross-matching	1
Surgical site not marked	1
Consent form not signed	0
Theatre x-ray scheduling conflict	2

**Table 3:** Comparison of the number of errors with and without the proforma Improvement in decreasing the errors.

	Errors causing a delay
Without proforma, n = 35	11 (31%)
With proforma, n = 30	5 (16%)

## Discussion

Orthopedics sees a high rate of patient turn-over, especially in trauma cases. Potential delays in the surgery schedule can disrupt the flow work in an operating theatre which can have widespread effects, especially in a busy tertiary care university hospital such as ours. The preparation errors we discovered in the first part of the audit were not extreme or difficult to correct, but the resultant delays have the potential for disastrous outcomes. The use of the proforma caused an increase in the number of fully prepared patients ready for surgery. That is, fewer patients experienced a delay-causing error in the preoperative period. The second leg of audit almost halved the patients coming to the theatre with missing documents or preparedness.

While our study was limited in that we used a relatively small sample size and short duration, our findings are still valid as a template for any future study with larger populations and longer durations. Other surgical specialities can adapt any similar proforma according to their own needs to help decrease the frequency of errors and delays.

## Conclusions

The added intervention of the proforma reduced the number of preoperative preparation errors. Health care facilities may benefit from a similar audit to assess error rates and improve operating theater efficiency, and while we intend to further improve the proforma developed in this study, it may prove useful for supplementing standardized, widely accepted preoperative protocols as an additional safety measure. A second study is planned with a larger survey sample that will use a modified proforma based on feedback received on the original proforma from this study.
